# *spaB*-positive *Erysipelothrix rhusiopathiae*, a novel teleost pathogen isolated from cultured barramundi

**DOI:** 10.1177/10406387231209035

**Published:** 2023-11-02

**Authors:** Ri K. Chang, Eric K. Pomaranski, Cem Giray, William Keleher, Matt J. Griffin, Alvin C. Camus, Kathy L. Toohey-Kurth, Esteban Soto

**Affiliations:** Department of Medicine & Epidemiology, School of Veterinary Medicine, University of California–Davis, Davis, CA, USA; Department of Medicine & Epidemiology, School of Veterinary Medicine, University of California–Davis, Davis, CA, USA; Kennebec River Biosciences, Richmond, ME, USA; Kennebec River Biosciences, Richmond, ME, USA; Department of Pathobiology and Population Medicine, College of Veterinary Medicine, Mississippi State University, Stoneville, MS, USA; Department of Pathology, College of Veterinary Medicine, University of Georgia, Athens, GA, USA; California Animal Health & Food Safety Laboratory, San Bernardino, University of California–Davis, Davis, CA, USA; Department of Medicine & Epidemiology, School of Veterinary Medicine, University of California–Davis, Davis, CA, USA

**Keywords:** barramundi, emerging infectious disease, *Erysipelothrix*

## Abstract

Members of the genus *Erysipelothrix* are emergent pathogens of cultured eels, as well as several characid and cyprinid species. Since 2013, *E. rhusiopathiae* has been reported from diseased barramundi (*Lates calcarifer*) cultured in North America; we recovered 8 *E. rhusiopathiae* isolates from diseased fish during different outbreaks from the same farm. The *E. rhusiopathiae* isolates from barramundi were compared phenotypically and genetically to *E. piscisicarius* isolates characterized from ornamental fish and *E. rhusiopathiae* recovered from aquatic and terrestrial animals. All barramundi isolates were PCR-positive for the surface protective antigen type B (*spaB*) gene, and shared ≥ 99.7% sequence similarity among concatenated multilocus sequence analysis gene sequences, indicating a high degree of genetic homogeneity. These isolates were > 99% similar to other *spaB-*positive isolates from marine invertebrates and marine mammals, consistent with findings for other *spa* types. The *spaA* and *spaB* isolates shared < 98% similarity, as well as < 90% similarity with *spaC-*positive *E. piscisicarius*. Similar clonality among the *spaB* isolates was observed using repetitive element palindromic PCR. In experimental intracoelomic injection challenges conducted to fulfill Koch postulates, 67% of exposed tiger barbs (*Puntigrus tetrazona*) died within 14 d of challenge. Our study supports previous work citing the genetic variability of *Erysipelothrix* spp. *spa* types and the emergence of members of the genus *Erysipelothrix* as nascent fish pathogens.

Barramundi (*Lates calcarifer*) are catadromous perciform fishes native to marine and freshwaters of the Indo-Pacific region and an increasingly popular aquaculture species with > 30,000 tons produced annually in Southeast Asia.^8^ Consistent with other aquaculture species, infectious disease causes significant economic losses in cultured barramundi. Several infectious agents have been reported from barramundi aquaculture, including, but not limited to, betanodavirus, *Vibrio* spp., *Aeromonas* spp., *Flavobacterium* spp., *Tenacibaculum maritimum*, and *Streptococcus iniae*.^6^

*Erysipelothrix* is a genus of *Firmicutes*, most often associated with disease in swine, turkey, sheep, and other terrestrial animals, and marine mammals.^16,20–22^ *E. rhusiopathiae* is typically regarded as a commensal of fish, although mortality in cultured eels (*Anguilla reinhardtii* and *A. australis*) associated with an *Erysipelothrix* sp. has been reported.^2^ A newly recognized member of the genus, *E. piscisicarius*, has been implicated in disease outbreaks in ornamental cyprinid and characid fish.^1,17–19^ Furthermore, *E. piscisicarius* has been observed in mosquitofish (*Gambusia affinis*) incidentally present in U.S. catfish aquaculture ponds, although without observed signs of disease.^23^

Phylogenetically, *E. rhusiopathiae* and *E. piscisicarius* cluster based on the isoform of their surface protective antigen (*spa*) gene. *E. rhusiopathiae* is associated with monophyletic groups possessing *spaA* and *spaB* genes; *E. piscisicarius* forms a genetically distinct lineage positive for the *spaC* isoform.^7,17–19,23^ Given the recent recognition of *E. piscisicarius*, and the lack of distinguishing phenotypic characters to differentiate between *E. rhusiopathiae* and *E. piscisicarius*, reports of *E. rhusiopathiae* from fish may be misleading.

A barramundi aquaculture farm in the United States reported increased losses associated with isolates consistent with *E. rhusiopathiae*, identified using newly established molecular techniques. We report here *spaB*-type *E. rhusiopathiae* causing disease in cultured barramundi.

## Materials and methods

### Fish history and preliminary diagnosis

Between 2013 and 2018, a barramundi aquaculture farm in the United States reported increased morbidity and mortality in all age classes cultured in a brackish water (4 g/L salinity) recirculating system. Initial diagnostic assessments attributed mortality to poor water quality and bacterial coinfections. During this period, clinically affected animals were submitted to Kennebec River Biosciences (Richmond, ME, USA) for diagnostic evaluation. Eight bacterial isolates were obtained from aseptically collected swabs of the brain and kidney from clinically affected barramundi during different outbreaks in 2013 (*n* = 1), 2016 (*n* = 2), and 2017 (*n* = 5). The isolates were cultured for 48 h at 30°C on trypticase soy agar supplemented with 5% sheep blood and 1.5% NaCl (blood agar; SBA). Individual colonies, annotated as BM1–7 and EryBarr (Table 1), were expanded in 5 mL of porcine brain heart infusion broth. Aliquots (1 mL) were prepared for cryopreservation by the addition of 15% v/v sterile glycerol and stored at −80°C. Isolates were initially identified as members of the genus *Erysipelothrix* by Gram stains and 16S rRNA gene amplification and sequencing by Kennebec River Biosciences.

**Table 1.** *Erysipelothrix* spp. isolates with their source information and *spa* type PCR amplification result used for multilocus sequence analysis comparison to barramundi (*Lates calcarifer*) *E. rhusiopathiae* isolates from this study.

Sample ID	Isolation location	Isolation source	*spa*
*Erysipelothrix rhusiopathiae*			
10506	IL, USA	Dolphin/blood	A
6567	IL, USA	Beluga/blood	A
H1T1	IL, USA	Feed/herring	A
9301985†	CA, USA	Elephant seal/lung	A
C1T0A	IL, USA	Feed/capelin	A
C1T0B	IL, USA	Feed/capelin	A
CAP2	IL, USA	Feed/capelin	A
CAHFS1†	CA, USA	Chicken/liver	A
CAHFS2†	CA, USA	Ovine/liver	A
CAHFS4	CA, USA	Caprine/liver	A
BM1† *	USA	Barramundi/kidney or brain	B
BM2†	USA	Barramundi/kidney or brain	B
BM2†	USA	Barramundi/kidney or brain	B
BM4†	USA	Barramundi/kidney or brain	B
BM5†	USA	Barramundi/kidney or brain	B
BM6†	USA	Barramundi/kidney or brain	B
BM7†	USA	Barramundi/kidney or brain	B
EryBarr†	USA	Barramundi/kidney or brain	B
DF-Kri B	IL, USA	Dolphin/feces	B
7122†	IL, USA	Beluga/blood	B
7155	IL, USA	Beluga/blood	B
262†	IL, USA	Dolphin/spleen	B
10792†	IL, USA	Beluga/blood	B
S4T0	IL, USA	Feed/squid	B
S1T0	IL, USA	Feed/squid	B
*Erysipelothrix piscisicarius*			
9711	IL, USA	Rasbora/kidney	C
14089A	IL, USA	Jewel tetra/kidney	C
15TAL0474	FL, USA	Ornamental fish	C
14TAL259A	FL, USA	Ornamental fish	C
14TAL259B*	FL, USA	Ornamental fish	C
14TAL259C	FL, USA	Ornamental fish	C
15TAL055U1	FL, USA	Ornamental fish	C
15TAL056U3	FL, USA	Ornamental fish	C
14TAL261U2	FL, USA	Ornamental fish	C
15TAL055K2	FL, USA	Ornamental fish	C
14TAL056U8	FL, USA	Ornamental fish	C
15TAL056K5	FL, USA	Ornamental fish	C
14TAL260U1	FL, USA	Ornamental fish	C
UGA21756	GA, USA	Ornamental fish	C
*Erysipelothrix tonsillarum*			
Q1†	IL, USA	Feed/squid	B

* Used for laboratory-controlled challenge.

† Used for repetitive element palindromic PCR.

### Phylogenetic characterization

A multilocus sequence analysis (MLSA) scheme was used to identify the barramundi isolates to species and compare them to other *Erysipelothrix* spp. isolates from fish, marine invertebrates, and marine mammals characterized in previous work (Table 1).^10,19^ Fragments of galactokinase (*galK*), glycerol-3-phosphate-dehydrogenase (*gpsA*), D-lactate dehydrogenase (*ldhA*), ribose-phosphate-pyrophosphokinase (*prsA*), phosphate acetyl-transferase (*pta*), adenylosuccinate synthetase (*purA*), recombinase A (*recA*), and DNA gyrase B (*gyrB*) genes were PCR-amplified,^19^ and purified amplicons were directly sequenced bidirectionally at the University of California-Davis–College of Biological Sciences–DNA Sequencing Facility (Davis, CA, USA). Sanger chromatograms were manually annotated and contiguous sequences for each gene fragment assembled in Geneious v.10.2.4.^11^ Reference gene fragments were concatenated for each isolate, the concatenates aligned with MUSCLE,^4^ and a maximum-likelihood tree generated using the Tamura–Nei model with 1,000 bootstrap replicates in MEGA v.7.^5,12,24^

The *spa* type for each barramundi isolate was determined as described previously.^21^ Briefly, genomic DNA (gDNA) was extracted (DNeasy blood and tissue kit; Qiagen). The *spa* type of each isolate was determined using a previously established multiplex end-point PCR capable of detecting and differentiating among *Erysipelothrix* spp. *spaA*, *spaB*, and *spaC* genes.^21^ Further genetic characterization was performed by repetitive element palindromic PCR (rep-PCR) following previously established protocols using the GTG5 primer.^9,26^ Dice similarity coefficients and unweighted pair group method with arithmetic mean (UPGMA) analysis of rep-PCR profiles were performed (Quantity One software v.4.6.9; Bio-Rad). Similarly, the presence of *E. rhusiopathiae* virulence factor genes was determined by multiplex PCR assay using protocols described previously.^10,18^

### Biochemical and proteomic analysis

Conventional biochemical characterization of 1 representative barramundi *E. rhusiopathiae* isolate (EryBarr) and 5 *Erysipelothrix* spp. isolates (4 *E. rhusiopathiae*, 1 *E. piscisicarius*) used for reference^19^ was performed at 30°C. Reactions included catalase, cytochrome oxidase, nitrate, esculin, indole, motility (Hardy), and urease (Remel).

Isolates were grown on 5% Columbia SBA (Remel) at 30°C for 48 h. Five well-isolated colonies for each isolate were extracted by suspending in 300 µL of deionized water and mixing well, then adding 900 µL of 100% ethanol (Sigma). The suspension was vortexed thoroughly, then centrifuged at 12,100 × *g* for 2 min at room temperature. Supernatant was decanted completely, and a second spin was done to remove residual alcohol. The pellet was air dried for 2–3 min, and 25 µL of 70% formic acid (LC-MS grade; Sigma-Aldrich) was added and the suspension was mixed carefully by pipetting 7–10 times followed by the addition of 25 µL of 100% acetonitrile (UHPLC grade > 99.9%; Sigma-Aldrich). After centrifugation for 2 min at 12,100 × *g*, 1 µL of the supernatant was spotted in duplicate onto the target plate, air dried, and then overlaid with the matrix, 2-cyano-3-(4-hydroxyphenyl) acrylic acid (HCCA; Bruker). The target plate was scanned in a MALDI Biotyper Sirius (Bruker), and resultant spectra were matched to profiles in the 2022 MBT Compass Library (research use only; Bruker) database.

### Laboratory-controlled tiger barb injection challenge

Because of the unavailability of barramundi for experimental study, virulence and pathogenicity challenges were performed in tiger barbs (TBs; *Puntigrus tetrazona*), which have been demonstrated as a fish species highly susceptible to *Erysipelothrix* spp. infections.^19^ Two hundred naïve TBs (~2 g each) were donated from the University of Florida–Tropical Aquaculture Laboratory (Ruskin, FL, USA). Two weeks before the challenges, 10 fish were euthanized with an overdose of sodium bicarbonate–buffered tricaine methanesulfonate (500 mg/L, MS-222; Syndel), and swabs of the brain and kidney were collected aseptically and cultured for 48 h at 30°C on SBA to ensure that fish were *Erysipelothrix* spp.–free. The remainder of the fish were acclimated in 130-L tanks holding ~90 L of fresh well water at 22 ± 2°C under flow-through conditions with constant aeration for 1 wk before challenge. The TBs were fed a commercial feed (TetraMin tropical flake feed; Tetra) at 1% of body weight daily.

Archived isolates (BM1 *spaB*-*E. rhusiopathiae* from barramundi and *E. piscisicarius* 14TAL259B) were revived from cryogenic storage on blood agar (72 h at 30°C), and colonies resuspended in PBS to an estimated concentration of 10^8^ CFU/mL via spectrophotometer. On the day of challenge, TBs were anesthetized in 100 mg/L buffered MS-222. Each fish was injected intracoelomically with 10^7^ CFU bacterial suspension/fish (100 µL of 10^8^ CFU/mL suspension) or 100 µL of PBS (negative controls; *spaB =* 15, *spaC =* 15, PBS = 15) and then added to a 19-L tank (*n* = 15 fish/tank) and maintained under conditions identical to the acclimation period. Fish were monitored twice daily for 21 d; severely moribund fish were euthanized following the same protocol as above. Freshly dead and euthanized fish had their brains cultured for bacterial re-isolation and then were fixed in 10% neutral-buffered formalin for histologic examination. Surviving fish were euthanized following the above protocol at the end of the trial and similarly cultured and fixed in formalin. Survival curves generated during laboratory-controlled challenges were compared using the log-rank test (α = 0.05) for statistical analysis.

### Histopathology

For light microscopic evaluation, the bodies of formalin-fixed TBs challenged with the barramundi isolate were demineralized in Kristensen solution for 2–3 h, serially trimmed into transverse sections throughout their body lengths, and placed into individually identified tissue cassettes. Tissues were processed routinely, and 5-µm sections were stained with H&E. Select sections were stained with a modified Brown and Hopps tissue Gram stain.

## Results

### Bacterial characterization

Bacterial cultures obtained from diseased barramundi were morphologically consistent with other isolates of *E. rhusiopathiae* on blood agar. Colonies were small (slightly larger than *E. piscisicarius*), spherical, and α-hemolytic. Maximum colony size was reached within 48 h at 30°C.

The 6 *Erysipelothrix* isolates had identical biochemical profiles except for the *E. piscisicarius* isolate, which was negative for H_2_S on triple sugar iron agar and SIM media, whereas the other isolates were positive (Table 2).

**Table 2.** Phenotypic and biochemical properties of an *Erysipelothrix rhusiopathiae* from a barramundi (*Lates calcarifer*, EryBarr), 2 *spaA E. rhusiopathiae* (6567, H1T1), 2 *spaB E. rhusiopathiae* (7122, 7155), and 1 *E. piscisicarius* (14TAL259B) isolates. All reactions carried out on sheep blood agar (SBA) unless otherwise noted.

Test/reaction	*E. rhusiopathiae*					*E. piscisicarius*
	EryBarr	6567	H1T1	7122	7155	14TAL259B
Growth in SBA at 30°C	+	+	+	+	+	+
Gram stain	+	+	+	+	+	+
Bacteria morphology	Short and long rods	Short and long rods	Short and long rods	Short and long rods	Short and long rods	Small coccoid rods
Hemolysis	α	α	α	α	α	α
Motility (SIM)	Non-motile	Non-motile	Non-motile	Non-motile	Non-motile	Non-motile
Catalase	–	–	–	–	–	–
Oxidase	–	–	–	–	–	–
NO_3_	–	–	–	–	–	–
Urease	–	–	–	–	–	–
Esculin	–	–	–	–	–	–
TSI	NC/NC acid base	NC/NC acid base	NC/NC acid base	NC/NC acid base	NC/NC acid base	NC/NC acid base
	H_2_S+	H_2_S+	H_2_S+	H_2_S+	H_2_S+	H_2_S–
SIM	Indole	Indole	Indole	Indole	Indole	Indole
	H_2_S+	H_2_S+	H_2_S+	H_2_S+	H_2_S+	H_2_S–
MALDI score	2.18, 2.21	2.31, 2.17	2.21, 2.14	2.21, 2.14	2.19, 2.19	1.73, 1.71

NC/NC = no change; SIM = sulfur indole motility medium; TSI = triple sugar iron agar.

*E. rhusiopathiae* had good spectral analysis scores (> 2.0; minimal 2.1 to maximal 2.31) indicating reliable identification to genus and species level for the isolates genotypically identified as *E. rhusiopathiae*. The scores of 1.73 and 1.7 for the *E. piscisicarius* duplicate spots allow identification to the genus level only, which was expected given that *E. piscisicarius* was not in the 2022 database.

Amplification and sequence analysis of the 16S rRNA and MALDI confirmed the isolates from barramundi to be *E. rhusiopathiae.* All isolates were PCR-positive for *spaB* and negative for other *spa* isoforms.

**Figure 1.**
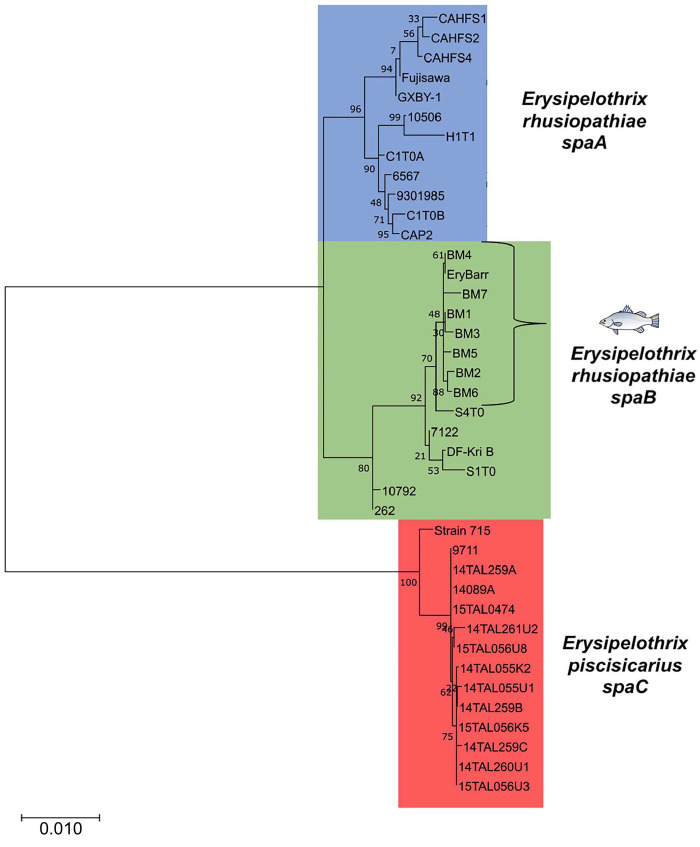
Maximum-likelihood phylogenetic tree of the multilocus sequence analysis (MLSA) gene sequences of *Erysipelothrix rhusiopathiae* isolates from clinically affected barramundi to other *Erysipelothrix* spp. isolates previously analyzed through MLSA (Table 1),^19^ including the reference strains Fujisawa (GenBank AP012027),^15^ GXBY-1 (CP014861.1),^25^ and *spaC*-positive strain 715 (PRJNA288715).^7^ Genes for MLSA were *galK*, *gpsA*, *ldhA*, *prsA*, *ptA*, *purA*, *recA*, and *gyrB*. Numerical values at nodes are the bootstrap values based on 1,000 pseudo-replicates, and the scale bar represents the number of substitutions per site.

In the MLSA-based phylogeny, the barramundi isolates clustered with other *spaB*-positive *E. rhusiopathiae* isolates characterized by previous investigations (Fig. 1; Table 1).^19^ Barramundi isolates appear largely clonal (> 99.7%; 6,239 of 6,250 nucleotides MLSA sequence identity) and most similar to a *spaB*-positive *E. rhusiopathiae* isolate from fish skin mucosa (S4T0; > 99% identity). Isolates were 97.6–98.0% identical in sequence to *spaA*-positive *E. rhusiopathiae* and only 89.6–89.9% similar to *E. piscisicarius* isolates.

**Figure 2.**
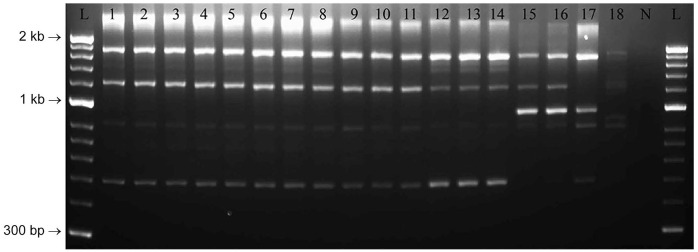
Agarose gel electrophoresis image of repetitive element palindromic PCR (rep-PCR) assay of *Erysipelothrix* spp. including the isolates from barramundi, and representative *spaA*-positive *E. rhusiopathiae*, *spaB*-positive *E. rhusiopathiae*, and *E. piscisicarius*. Lanes: 1–7 = BM1–7, respectively; 8 = EryBarr; 9–11 = *spaB*-positive *E. rhusiopathiae* from clinically affected marine mammals (9 = 262, 10 = 7122, 11 = 10792); 12–14 = *E. piscisicarius* from clinically affected fish (12 = 14TAL260U1, 13 = 15TAL055U1, 14 = 15TAL055K2); 15–17 = *spaA*-positive *E. rhusiopathiae* from clinically affected mammals and birds (15 = CAHFS1, 16 = CAHFS2, 17 = 9301985); 18 = *E. tonsillarum*; N = no template control; L = 50-bp ladder.

**Figure 3.**
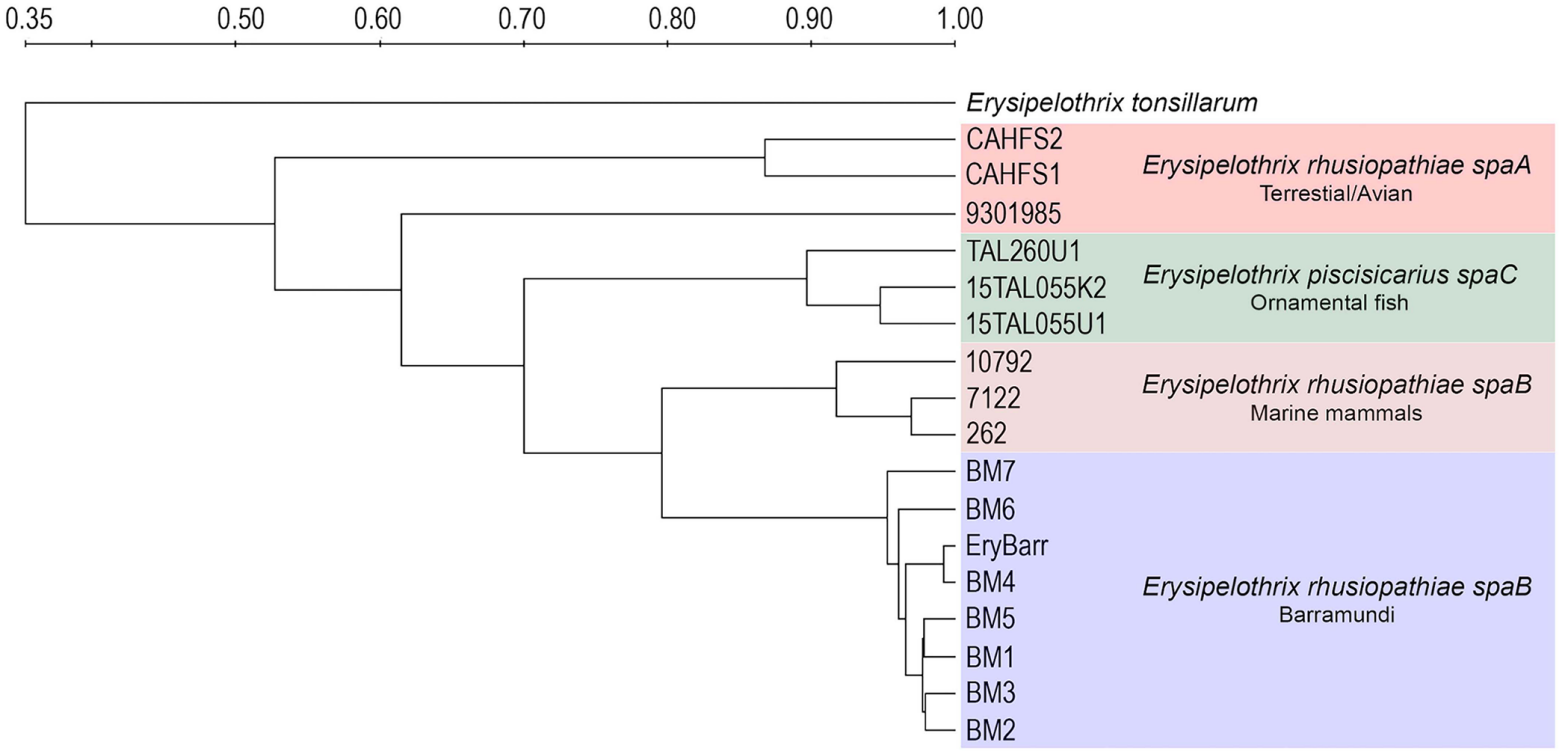
UPGMA dendrograms based on Dice coefficient matrices generated from PCR amplification of *Erysipelothrix* spp. DNA using the GTG5 primer.^9,26^ The *spaB*-positive *E. rhusiopathiae* isolates from barramundi formed a distinct cluster with other *spaB* isolates, separate from archived *E. piscisicarius*, *spaA*-positive *E. rhusiopathiae* from terrestrial animals, and *E. tonsillarum*.

Rep-PCR profiles had consistent molecular fingerprints for the isolates from barramundi and other *spaB*-positive isolates from diseased marine mammals (Fig. 2). Based on Dice similarity coefficients and UPGMA analysis, profiles for *spaB*-positive isolates formed a discrete cluster separate from *spaA E. rhusiopathiae* and *E. piscisicarius* isolates from various hosts (Fig. 3).

Virulence factor profiles for *spaB-*type *E. rhusiopathiae* from barramundi were similar to other *spaB-*type *E. rhusiopathiae* believed to be commensals in fish (isolates S4T0 and S1T0). Isolates recovered from barramundi and isolate S1T0 were all PCR-positive for the following virulence factor genes: ABC transporter metal-binding protein (ERH_136), alginate-O-acetyltransferase (*algI*), *rhusiopathiae* surface protein (*rspB*), the *cap locus*, neuraminidase (*nanH*), superoxide dismutase (*sodA*), heparinase (*hep*), fibronectin-binding protein (*fbpA*), hyaluronidase (*hylA*), and membrane attachment protein (*dnaB*). With the exception of *algI*, isolate S4T0 had a similar virulence factor gene profile.

### Laboratory-controlled tiger barb injection challenge

**Figure 4.**
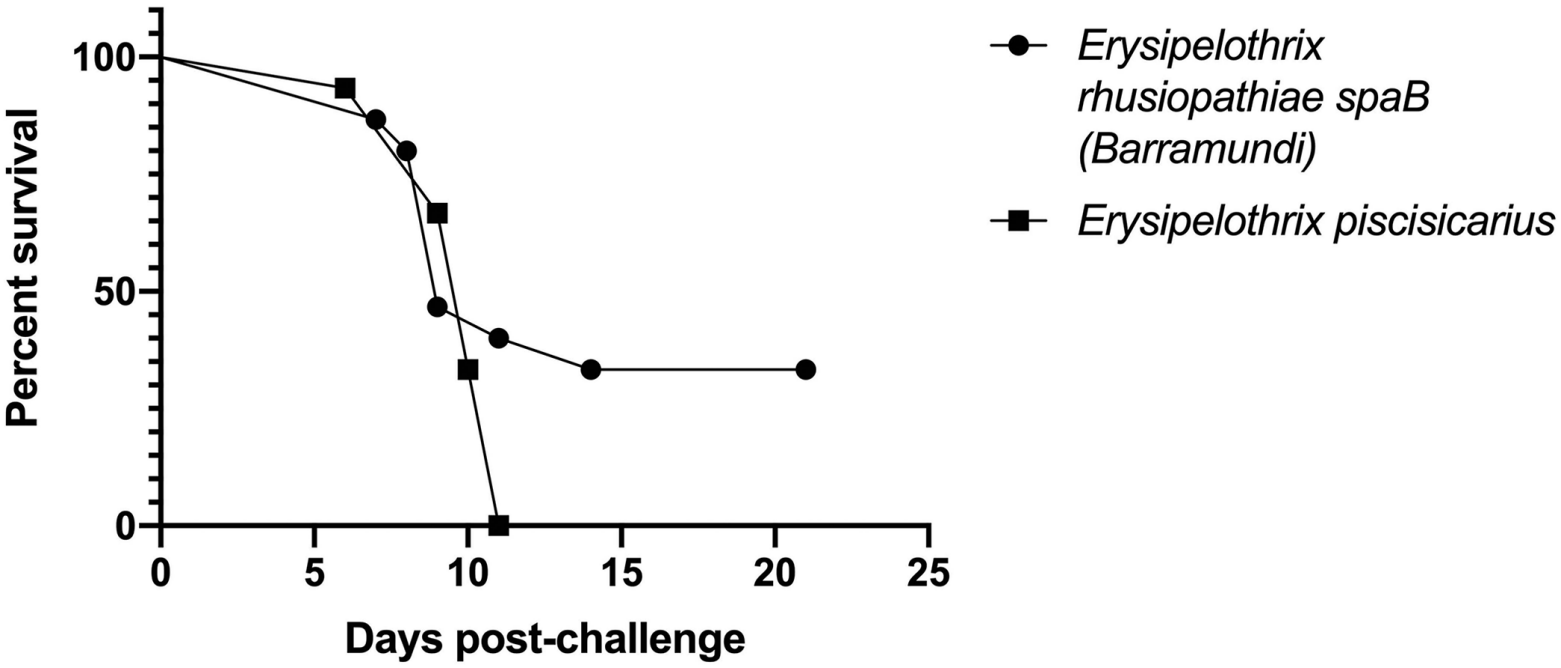
Percent survival from laboratory-controlled intracoelomic injection challenges in the tiger barb (*Puntigrus tetrazona*) infection model. Fish were injected with 100 μL of 10^8^ CFU/mL (10^7^ CFU/fish) *spaB*-positive *Erysipelothrix rhusiopathiae* (BM1) or *spaC*-positive *E. piscisicarius* from clinically affected ornamental fish (14TAL259B).

TBs exposed by intracoelomic injection to the *spaB*-positive isolate from barramundi suffered 67% mortality by day 14; *E. piscisicarius* produced 100% mortality, including 4 moribund fish that were euthanized (Fig. 4). There was no significant difference between the 2 curves as determined by log-rank (Mantel–Cox) test (*p* = 0.183). The *spaB* barramundi isolate was successfully re-isolated from 6 of 10 TB-challenged mortalities. Bacteria isolated from the brains of moribund fish were confirmed to be *E. rhusiopathiae* using the *spa* multiplex PCR assay.^21^ Similarly, *spaC* isolates were recovered from 4 of 15 fish challenged with *E. piscisicarius*. There was no bacterial growth on SBA from brain cultures of fish (i.e., controls and challenged survivors) euthanized at the end of the trial.

### Histopathology

**Figure 5.**
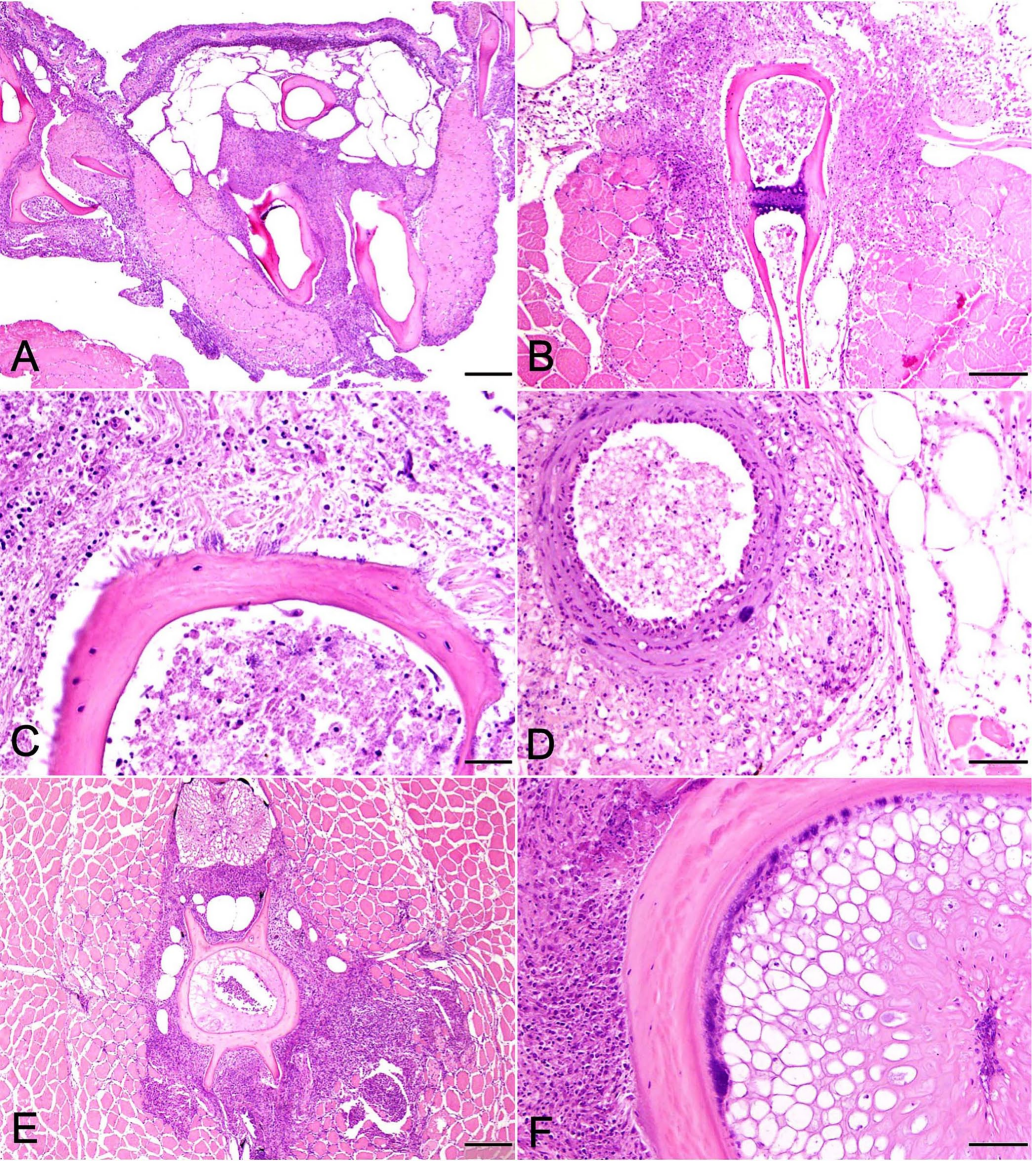
Pathologic changes in tiger barbs (*Puntigrus tetrazona*) challenged by intracoelomic injection with a *spaB*-positive *Erysipelothrix rhusiopathiae* isolate (BM1) from barramundi (*Lates calcarifer*). **A.** Pyogranulomatous inflammatory cell infiltrate and necrotic debris surround bone and extend into adjacent connective tissues within the intermandibular (isthmus) region 8 d post-challenge. H&E. Bar = 200 µm. **B.** Inflammatory infiltrates and necrotic debris surround bone and fill interosseous spaces within dorsal fin pterygiophores 9 d post-challenge. Inflammation extends into endomysial areas of adjacent skeletal muscle, isolating and replacing scattered myofibers. H&E. Bar = 100 µm. **C.** Higher magnification of Fig. 5B demonstrating slender bacterial rods palisading from the bone surface. H&E. Bar = 20 µm. **D.** Ventral aorta with bacterial colony in the medial tunic 11 d post-challenge. H&E. Bar = 50 µm. **E.** Intense perivertebral granulomatous inflammation and fibroplasia replacing skeletal muscle in fish surviving the 21-d challenge. H&E. Bar = 200 µm. **F.** Masses of bacteria sequestered within the intervertebral notochord and covering the bone of a vertebral centrum in 21-d challenge survivor. H&E. Bar = 50 µm.

Fish challenged with the barramundi *E. rhusiopathiae* isolate that died 7–8 d post-injection challenge had similar inflammatory lesions localized to the mandibular region and various fin bases (Fig. 5A, 5B). Bone surfaces, periosteal fibrous tissue, and adjacent adipose tissue were infiltrated by moderate to occasionally severe pyogranulomatous infiltrates, dominated by macrophages with smaller numbers of neutrophils and scattered lymphocytes, accompanied by multifocal areas of necrotic cellular debris. Inflammatory infiltrates occasionally spilled into interstitial areas of adjacent skeletal muscle (Fig. 5B). Thin, gram-positive bacterial rods were first visualized in the day 7 sample, becoming more numerous by day 9, in association with bone surfaces or lying parallel to adjoining collagen fibers (Fig. 5C).

Lesion distribution varied between individuals but was more widespread in fish collected on day 9. Lesions remained focused on bone, particularly cranial bones, some of which developed eroded-to-scalloped margins indicative of osteolysis. Inflammatory infiltrates were subjectively more intense, and bacterial numbers were slightly greater. In 1 or 2 fish each, lesions involved opercular bones, vertebral bodies, and vertebral spines. Scattered scale pockets contained edema fluid mixed with minimal numbers of neutrophils and necrotic debris. Skin surfaces overlying periosteal lesions in the mandibular region and opercula, as well as the pharyngeal mucosa, were often eroded-to-ulcerated and infiltrated by scattered lymphocytes but no bacteria. In 2 fish, inflammatory infiltrates and rare bacterial colonies extended from the intermandibular region replacing muscle and adipose tissue along the aorta and bulbus arteriosus (Fig. 5D). In one barb, macrophages extended from perivertebral bone to circumferentially infiltrate the swim bladder adventitia.

**Figure 6.**
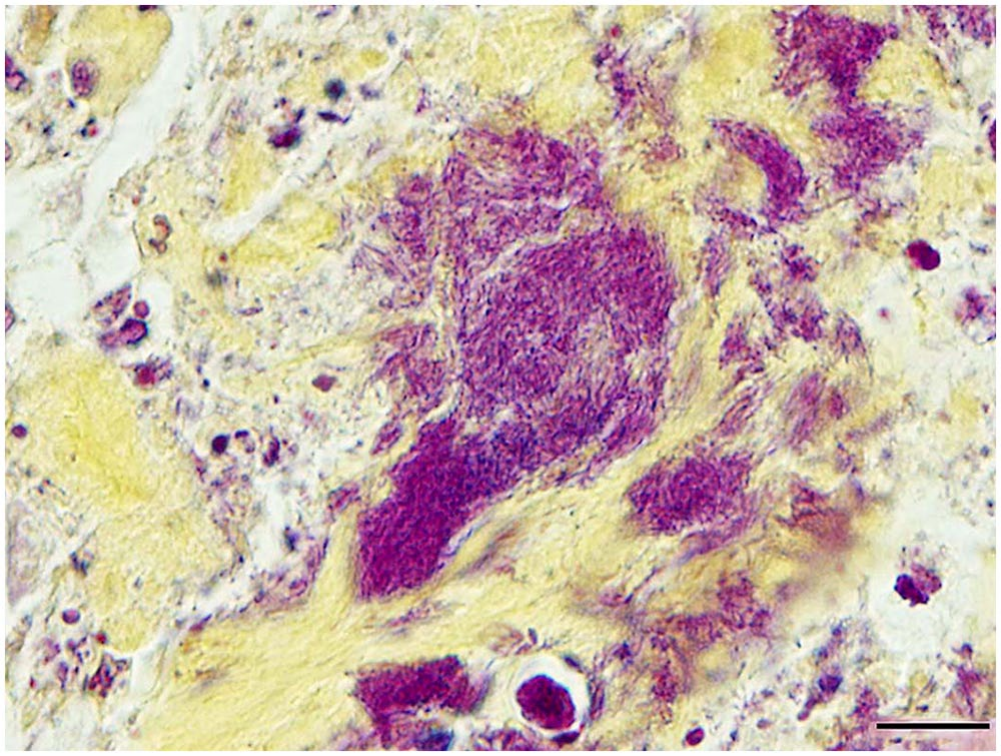
Gram-positive bacterial rods consistent with an *Erysipelothrix* sp. within notochordal remnants of a chronically infected tiger barb (*Puntigrus tetrazona*) challenged with a *spaB*-positive *Erysipelothrix rhusiopathiae* isolate from barramundi (*Lates calcarifer*). Brown and Hopps. Bar = 10 µm.

Pathologic changes followed a similar course and distribution as above in fish from days 11 and 14. By day 21, inflammatory cell infiltrates in euthanized surviving fish surrounded bone and were dominated almost entirely by sheets of macrophages, scattered lymphocytes, and mild fibroplasia. In 2 of 3 fish, inflammatory changes surrounding vertebral bone were severe, radiating into adjacent muscle interstitia or replacing areas of skeletal muscle entirely (Fig. 5E) In these fish, bacteria were only observed in association with vertebral bone and intervertebral notochord (Fig. 5F). Consistent with an *Erysipelothrix* spp., the bacterial rods stained gram-positive (Fig. 6). Small, regenerating foci of dysplastic cartilage were present in the cranial bone of one fish. Congestion and mild inflammation surrounded the swim bladder of one TB, and severe pyogranulomatous coelomitis involved a second. No bacteria were observed in either.

## Discussion

*Erysipelothrix rhusiopathiae* is the causative agent of erysipelas in mammals and erysipeloid in humans, but until 2014 was not thought to cause disease in fish.^14,20^ However, reports have implicated one or more *Erysipelothrix* spp., including *E. rhusiopathiae*, with disease outbreaks in several fish species.^2,18,23^ To our knowledge, *spaB*-positive isolates of *E. rhusiopathiae* have not been reported to cause disease in barramundi. Based on MLSA and rep-PCR analysis, the barramundi isolates were largely clonal and shared a similar virulence factor gene profile to other *spaB*-positive *E. rhusiopathiae* isolates reported as commensals in fish. The clonality of the barramundi isolates is unsurprising given that they were all recovered from the same farm. Previous studies indicate that *spaB*-positive *E. rhusiopathiae* were avirulent in TBs.^19^ Given that these isolates had comparable virulence factor gene profiles, the mortality differences may be attributable to the different routes of infection (immersion vs. intracoelomic injection), but unidentified virulence factor genes in the barramundi isolates could also have contributed.

Our in vivo challenge with a *spaB*-positive isolate from barramundi demonstrated virulence in TBs by intracoelomic injection. Intracoelomic and immersion methods of challenge have been validated for *E. piscisicarius* in zebrafish (*Danio rerio*), Nile tilapia (*Oreochromis niloticus*), and TBs.^1,17–19^ Intracoelomic exposure routes typically result in greater mortality and successfully induce lesions characteristic of naturally occurring infections.^18^ Furthermore, intracoelomic challenges also have advantages over immersion challenges, including standardization of inoculum and predictable induction of acute infection, because the bacteria can bypass the fish’s natural defenses of mucus and epithelium.^13,27^ Although immersion challenges may better mimic natural routes of infection, they tend to have more variable morbidity and mortality rates and require higher concentrations of bacteria, which can confound pathology assessments.^3,18^ However, future studies evaluating the pathogenesis of this *E. rhusiopathiae* isolate via immersion challenge are warranted.

Although not all challenged fish were in suitable condition for evaluation because of rapid autolysis in water, histopathologic findings provide insight into aspects of disease pathogenesis including bacterial tissue and site preferences, lesion progression, and associated inflammatory responses. Similar to *E. piscisicarius*, the *spaB*-positive *E. rhusiopathiae* exhibited distinct tropism for connective tissues. However, unlike *E. piscisicarius*, our *E. rhusiopathiae* isolate was never observed in large numbers and was limited to periosteal fibrous connective tissues and bone surfaces. The massive colonization of dermal connective tissue and muscle interstitia characteristic of *E. piscisicarius* infection was not observed, and bacteria did not course within vascular walls to reach the coelomic viscera.^18,19^ We observed bacteria only once in association with a vascular wall and in sparse isolated colonies.

Early in the course of infection, and despite intracoelomic injection, lesions were limited to peripheral bones of the mandibular region and fin bases but spread centrally to involve multiple cranial bones, including the opercula. Vertebral bodies and spines were the final sites of lesion progression, which after 2–3 wk manifested as severe lesions in multiple fish. Concomitant with chronicity, bacterial numbers declined as inflammatory responses intensified and became increasingly dominated by macrophages and fibroplasia. Similar to *E. piscisicarius*, bacteria appeared to persist within the intervertebral notochord, in which inflammatory responses were often minimal or nonexistent.^18^

We identified a *spaB-*positive isolate of *E. rhusiopathiae* as a novel pathogen of cultured barramundi to be added to the list of *Erysipelothrix* spp. emerging as disease agents in fish. Moreover, our results highlight distinctions between commensalism and pathogenicity, raising questions about how genetically similar bacteria can produce variable responses in different fish hosts. Future studies investigating the transcriptional and genetic factors responsible for these differences will shed light on the complex interactions between these agents and their hosts, as well as provide information beneficial to aquaculture and associated industries attempting to understand and control these emergent pathogens.
